# Spatiotemporal dynamics of age-related genes and the regulatory network of *LaAGL2-3* in *Larix kaempferi* (Lamb.) Carr. based on the latest genome annotation

**DOI:** 10.1186/s12870-025-07956-y

**Published:** 2025-12-15

**Authors:** Tang-Quan Liao, Ao-Jie Luo, Zha-Long Ye, Wanfeng Li

**Affiliations:** 1 State Key Laboratory of Tree Genetics and Breeding, Key Laboratory of Tree Breeding and Cultivation of the National Forestry and Grassland Administration, Research Institute of Forestry, Chinese Academy of Forestry, Beijing, 100091 People’s Republic of China; 2 Department of Plant Physiology, Umeå Plant Science Centre (UPSC), Umeå University, Umeå, 90187 Sweden

**Keywords:** Tree aging, Conifer, Age-related gene expression, MADS-box transcription factor, Spatiotemporal regulation, AGL6, Transcriptomics.

## Abstract

**Background:**

In plants, time information is recorded in strictly ordered sequences of development events, resulting in age-associated physiological and morphological differentiation, including clear spatial gradients from the base to the crown within a single tree. However, the molecular mechanisms driving such ontogeny-related differentiation remain largely unknown.

**Results:**

Based on our newly generated *Larix kaempferi* (Lamb.) Carr. genome annotation, we identified 307 age-related genes, which were grouped into two expression clusters reflecting opposite temporal trends. Spatial expression analysis further revealed 13 differentially expressed genes along the vertical axis of the tree, suggesting their roles in regulating spatially distinct physiological traits. Yeast one-hybrid and dual-luciferase reporter assays demonstrated that *LaAGL2-3b* directly binds to the promoters of six genes, including *LaAGL2-3a*, *LaAGL2-3b* (self-regulation), and *L. kaempferi cycloartenol synthase* (*LkCAS1*). Over-expression of *LaAGL2-3* in *Arabidopsis thaliana* (L.) Heynh. significantly accelerated life cycle progression, supporting its functional involvement in aging-related developmental processes.

**Conclusions:**

Our results indicate that *LaAGL2-3* shows coordinated temporal and spatial expression dynamics with other age-related genes in *L. kaempferi*. This coordinated pattern offers hypotheses about its potential role within age-associated regulatory processes. The genomic and transcriptomic resources generated here offer a foundation for future functional investigations and for improving our understanding of conifer development and ontogeny.

**Supplementary Information:**

The online version contains supplementary material available at 10.1186/s12870-025-07956-y.

## Background

In the life cycle of plants, development events occur in a strict sequence. A seedling starts vegetative growth after seed germination and then enters the reproductive phase, in which it can flower and produce seeds. For *Larix kaempferi* (Lamb.) Carr., about 10 years is required for one life cycle to finish, whereas 20 years is needed for *Picea abies* (L.) H. Karst. In this dynamic occurrence of life cycle events, time information is recorded and kept, resulting in variant age-dependent physiological characteristics. For example, juvenile seedlings often exhibit faster growth rates and extended growth periods compared to mature trees [1, 2]. Meanwhile, as trees grow, they exhibit an age gradient from the base to the crown, resulting in distinct and spatially distributed phenotypic and physiological traits, including wood properties, leaf morphology, protein identity, and regeneration capacity of vegetative propagation [3–7]. Despite these well-documented observations, the molecular mechanisms underlying these age-related differences in trees and their interconnections across temporal and spatial dimensions remain poorly elucidated.

A growing body of research has identified numerous genes that regulate plant aging, with distinct roles in promoting or delaying developmental transitions. Genes such as *Suppressor of Overexpression of Constans 1* (*SOC1*) [8], *APETALA1* (*AP1*) [9], *Squamosa-Promoter Binding Protein-Like* (*SPL*) [10, 11], and *DAL1* (a MADS-box gene) [12] drive maturation, while microRNAs such as miR156 [13] and miR171 [14] and genes like *TERMINAL FLOWER1* (*MdTFL1* and *MdTFL1a*) [15], *Juvenile-to-Adult transition* (*JAT*) [16] and *LaAP2L1* (a heterosis-associated AP2/EREBP transcription factor from *Larix*) [17] maintain juvenility or delay maturation. Some of these genes have been proposed as molecular markers for assessing plant aging states, which allows us to determine the age status of trees in practice. For instance, miR156 serves as a marker of juvenility [13, 18], while *DAL1* and *SOC1* may reflect broader life-cycle stages [7, 19, 20]. In slash pine (*Pinus elliottii × caribaea*), the age-marking genes, including *PtAP2L3*, *PtDAL10* and *PtMADS28*, have been successfully used to elucidate the spatial patterns of ontogenetic ageing [21]. Although these discoveries have been well-documented in developmental transitions, little is known about how these age-related genes regulate phenotypic and physiological variations in space.

Previously, a comparative transcriptomic analysis was performed in *L. kaempferi* to reveal the molecular basis of age-dependent development processes. Twenty-seven age-related transcription factors differentially expressed between the juvenile vegetative phase and the adult reproductive phase were identified through transcript assembly [22]. Among them, *LaDAL1*, a MADS-box transcription factor and a homolog of *Arabidopsis thaliana* (L.) Heynh. *AGL6*, was found to coordinate with age and environmental signals in the life cycle of *L. kaempferi* and to accelerate the life cycle progression of *A. thaliana* by promoting the transitions of meristem fate [19, 20]. Furthermore, the genome assembly of *L. kaempferi* was revealed [23, 24], providing a genome reference for easier and more precise investigations of conifer trees’ biological function compared to transcript assembly. So far, various studies have investigated the age-dependent expression patterns and functions of age-related transcription factors. However, further research of the underlying regulatory mechanism and function of other potential age-related genes is still needed, especially when working with the reference genome.

Here, we identified age-related genes from previous age-series samples [25] using our new structure annotation of the latest genome of *L. kaempferi*, analyzed their potential functions and investigated their spatial expression patterns using our new spatial samples. Then, we screened out 13 differentially expressed age-related genes in spatial dimensions, including the MADS-box transcription factor *LaAGL2-3b*, which was identified as an age-related gene in our previous study [7]. Further, yeast one-hybrid (Y1H) and dual-luciferase reporter (DLR) assays confirmed direct regulatory relationships between *LaAGL2-3b* and six other genes. Additionally, over-expression of *LaAGL2-3* accelerated development transitions in *A. thaliana*, suggesting that it may play a role in regulating life cycle progression. These findings expand the range and function of multidimensional age-related genes in *L. kaempferi* and provide new targets for promoting vegetative transitions, offering theoretical support for studying the mechanism of age-dependent phenotypic and physiological variations in plants.

## Methods

### Sample preparation and sequencing

Age-series samples were collected and sequenced in our previous study [25]. In July 2011, we harvested the current-year uppermost main stems, the newly formed terminal shoot of the main stem produced during the current growing season, from *L. kaempferi* trees aged 1, 2, 5, 10, 25, and 50 years. All needles and branches were removed from each stem. For every age group, stems from at least three individual trees with comparable vigor were collected and pooled to generate one representative sample.

To examine vertical variation, spatial (height-gradient) samples were collected from the same three 25-year-old trees as those listed above and sequenced in the present study. After each tree was felled and the current-year uppermost main stem was sampled as an age-series sample, the left main stem was divided into three sections corresponding to (1) the lower part (D): approximately 0.40 m above ground; (2) the middle part (M): approximately 8.93 m above ground; and (3) the upper part (U): approximately 17.85 m above ground. At each of the three height positions, a 10–15 cm trunk segment was cut for sampling, resulting in three biological replicates per height. For each segment, the bark was removed and tissues for RNA extraction were collected by scraping the surface of the xylem with a sterile scalpel.

All collected samples were immediately frozen in liquid nitrogen and stored at − 80 °C until RNA extraction. RNA extraction, cDNA library construction, and Illumina sequencing followed the same standardized protocols as those described in our previous work [26].

The trees were planted in the State-owned Dagujia Forestry Farm (42°22′ N, 124°51′ E) in Qingyuan Man Autonomous County, Liaoning Province, Northeast China. The species *L. kaempferi* is widely cultivated in this farm, and its identity was confirmed based on morphological characteristics and local forestry records by Yue Wang and Yudong Ren, staff members of the State-owned Dagujia Forestry Farm.

### Genome structure annotation

The recently published genome assembly of *L. kaempferi* (NCBI GenBank accession number: GCA_027924585.1) [24], with a total size of 13.5 Gb (Table S1), was used for structure annotation.

We first used RepeatModeler v2.0.5 to generate a de novo repeat library for the genome, integrating TRF, RepeatScout, and RECON [27–29] with default settings. The resulting custom repeat library was then supplied to RepeatMasker v4.1.6, which was run with default parameters to annotate and mask repetitive elements, including retrotransposons, DNA transposons, and tandem repeats. The repeat-masked genome produced by this step was subsequently used as the input for downstream gene prediction.

Structural annotation was performed using BRAKER3 [30–33], a fully automated pipeline that integrates de novo prediction, protein homology, and transcriptome evidence (Figure S1). In this step, three data sources were provided as the input of BRAKER3: (1) the repeat-masked genome assembly, (2) the RNA-seq datasets generated in our lab, and (3) a combined protein database derived from previously published predictions [23, 34]. BRAKER3 was run in RNA-seq + protein data mode using default parameters. 

Briefly, RNA-seq reads were aligned to the genome using HISAT2 [35] and assembled with StringTie2 [36]. The resulting transcripts were used for initial gene prediction with GeneMarkS-T [37], followed by homology-based refinement using ProtHint [32]. BRAKER3 iteratively combined these data to train GeneMark-ETP and AUGUSTUS [38–40], and the final gene models were selected using TSEBRA [41]. After structural annotation, we assessed the completeness of our genome annotation using the BUSCO (Benchmarking Universal Single-Copy Orthologs; http://busco.ezlab.org/) [42] v6.0.0 pipeline with the 1,614 embryophyte_odb10 BUSCO genes as the reference.

Non-coding RNA, a kind of RNA with various known functions, including microRNA (miRNA), ribosomal RNA (rRNA), transfer RNA (tRNA), and other RNA families, was predicted using Infernal v1.1.5 [43] with Rfam database v14.10 [44] under default parameters. All predicted non-coding RNA features were converted to GFF3 format for downstream analysis.

The predicted genes were functionally annotated using the BLAST toolkit against seven databases: NR (NCBI nonredundant protein sequences), UniProt (the Universal Protein knowledgebase), Pfam (Protein family), GO (Gene Ontology), KEGG (Kyoto Encyclopedia of Genes and Genomes), eggNOG (Evolutionary Genealogy of Genes: Non-supervised Orthologous Groups) and PlantTFDB (Plant Transcription Factor Database). The searching parameters were “--evalue 1e-5 --max-target-seqs 1 –sensitive”.

### Transcriptome quantification

To generate a gene expression matrix, a reference-guided transcriptomic workflow was implemented. Raw sequencing reads were first processed using fastp v0.23.4 [45] to remove adapters, low-quality bases, and ambiguous reads. Cleaned reads were then aligned to the *L. kaempferi* reference genome [24] using HISAT2 v2.2.1 [35]. Alignment statistics were assessed using SAMtools v1.13 [46]. Gene-level quantification was performed with featureCounts v2.0.6 [47] using our newly generated structural genome annotation. Transcript abundance was normalized using the TPM (Transcripts Per Million) method to allow cross-sample comparison. All steps above were compiled into a workflow using Snakemake [48] for reproducibility and pipeline efficiency [49].

### Identification of age-related genes

To identify age-related genes, we assumed that gene expression patterns change systematically over growth time. Three complementary statistical methods were applied to screen candidate genes: time trend analysis (TTA), Spearman correlation analysis (SCA), and generalized linear modeling analysis (GLMA).

First, TTA was performed using the Mfuzz R package to identify genes exhibiting consistent temporal expression trends across the age gradient. Second, SCA was conducted to assess the monotonic association between gene expression and sample age (Table S2), with significance determined at *p* < 0.05. Third, GLMA was applied using the limma package with a linear time-series design, identifying genes with age-associated expression dynamics at a liberal threshold of *p* < 0.5.

Only genes that passed all three methods were retained as robust age-related candidates. This intersection approach was used to determine final age-related genes. All analyses were conducted using R v4.3.2.

### Functional enrichment analysis (GO and KEGG)

GO and KEGG enrichment analyses were performed to investigate the functional categories and pathways associated with age-related genes. Both analyses were conducted using the ClusterProfiler R package v4.10.0 [50] with default parameters.

### Spatial expression analysis of age-related genes

To assess the spatial expression patterns of age-related genes, expression data were extracted from height-gradient (D–M–U) samples. Genes with zero or highly unstable expression levels were excluded, based on the following criteria: TPM standard deviation > 50 within sample groups or TPM = 0 across all samples. The genes that passed this filtering step were subjected to hierarchical clustering using the ward.D2 method to group genes with similar spatial expression patterns. Differential expression analysis was performed using edgeR v4.0.16 [51]. Pairwise comparisons between stem segments (“D vs. M” and “M vs. U”) were used to identify differentially expressed genes (DEGs), with thresholds set at *p* < 0.05 and |log₂FC| > 0.5. Only genes that were differentially expressed in at least one of the two comparisons were considered for further analysis.

### Cloning of full-length cDNAs and promoter regions

Branches from 29-year-old *L. kaempferi* trees were collected in February 2023 for RNA extraction. Lateral branches located on the uppermost portion of the main stem were selected. After removing all buds, the remaining stems were used. Each branch segment was 14 cm in length. The trees were planted in the State-owned Dagujia Forestry Farm. Total RNA was extracted using the EasyPure^®^ Universal Plant Total RNA Kit (ER302-01, TransGen Biotech, Beijing, China), following the manufacturer’s instruction. First-strand cDNA synthesis was performed with 2.0 µg of total RNA using the TransScriptOne-Step gDNA Removal and cDNA Synthesis SuperMix (AH311-03, TransGen Biotech). The resulting cDNA was diluted and used as a template for cloning. Gene-specific primers were designed based on transcript sequences from the genome annotation (Table S3) to amplify full-length cDNAs. PCR products were purified with the EasyPure^®^ Quick Gel Extraction Kit (EG101-02, TransGen Biotech), ligated into the pEASY^®^-T1 cloning vector (CB501-02, TransGen Biotech), and verified by Sanger sequencing.

Genomic DNA was extracted from the same branches using the Hi-DNAsecure Plant Kit (DP350-03, TIANGEN Biotech, China). Promoter sequences (2,000 bp upstream of the start codon) were obtained from the genome annotation. Specific primers were designed for promoter amplification (Table S3), and PCR products were purified and cloned using the same methods as described for cDNA cloning.

### Promoter binding site prediction

Promoter sequences of the selected age-related genes were submitted to the PlantRegMap database [52] to predict potential transcription factor binding sites. Predictions were based on known transcription factor binding motifs from four model species: *A. thaliana*, *Oryza sativa* subsp. *japonica*, *P. abies* and *Populus trichocarpa* Torr. & A.Gray ex Hook.

### Yeast one-hybrid assay for promoter–transcription factor (TF) interactions

Coding sequences of candidate transcription factors were amplified by PCR and cloned into the pGADT7 vector to generate prey constructs (pGADT7–TF) [53] (Table S3). Promoter fragments of target genes were amplified and inserted into the pHIS2 vector to generate bait constructs (pHIS2–promoter) [53] (Table S3).

Prey and bait plasmids were co-transformed into *Saccharomyces cerevisiae* strain Y187 using the lithium acetate method [53]. Transformants were first selected on SD/–Leu/–Trp medium and incubated for 3–5 days. Positive clones were then transferred to SD/–Leu/–Trp/–His medium supplemented with 30, 40, or 50 mM 3-amino-1,2,4-triazole (3-AT) to assess promoter–TF interactions. Protein–DNA binding was inferred from yeast colony growth under selective conditions.

### Dual-luciferase reporter assay in larch callus

Full-length coding sequences of transcription factors were cloned into the pGreenII 62-SK vector to generate effector constructs (Table S3). Promoter sequences of the target genes were inserted into the pGreenII 0800-LUC vector to generate reporter constructs (Table S3). All constructs were introduced into *Agrobacterium tumefaciens* strain GV3101 [53].

*A. tumefaciens* cultures carrying effector and reporter plasmids were co-infiltrated into *L. kaempferi* callus tissues [54]. After incubation, firefly luciferase (LUC) and Renilla luciferase (REN) activities were measured using the Dual-Luciferase^®^ Reporter Assay System (Promega, USA). Promoter activity was represented by the LUC/REN ratio. For each effector–reporter combination, measurements were obtained from at least three independent biological replicates.

### Functional validation in transgenic *A. thaliana*

Seeds of *A. thaliana* ecotype Columbia-0 (Col-0) were surface-sterilized with 0.9% NaClO solution and germinated on half-strength Murashige and Skoog (½ MS) medium. After stratification at 4 °C for three days, seedlings were grown under a 16 h light/8 h dark photoperiod at 22 °C and 60% relative humidity. When seedlings developed 2–3 true leaves, they were transplanted into pots containing a 1:1 mixture of nutrient soil and vermiculite.

The full-length coding sequence of *LaAGL2-3b* was cloned into the pK2FMCS7 binary vector under control of the CaMV 35 S promoter, generating the construct 35 S::*LaAGL2-3b*. Transformation was carried out using the floral dip method with *A. tumefaciens* strain GV3101. Transgenic lines were selected on kanamycin-containing (50 mg/L) LB agar plates. Homozygous T3 plants were verified by PCR and used for phenotypic analysis.

Phenotypic traits assessed included bolting time, first flowering time, the time of formation of the last flower in the principal inflorescence axis, the number of rosette leaves and branches, and the length of the inflorescence axis. Measurements were performed on at least 10 independent plants per line. The Wilcoxon test was used to determine whether there was a significant difference between wild-type and other groups. All statistical analyses and visualizations were conducted using the tidyverse package in R.

## Results

### Annotation of genome structure and gene function

**Fig. 1:**
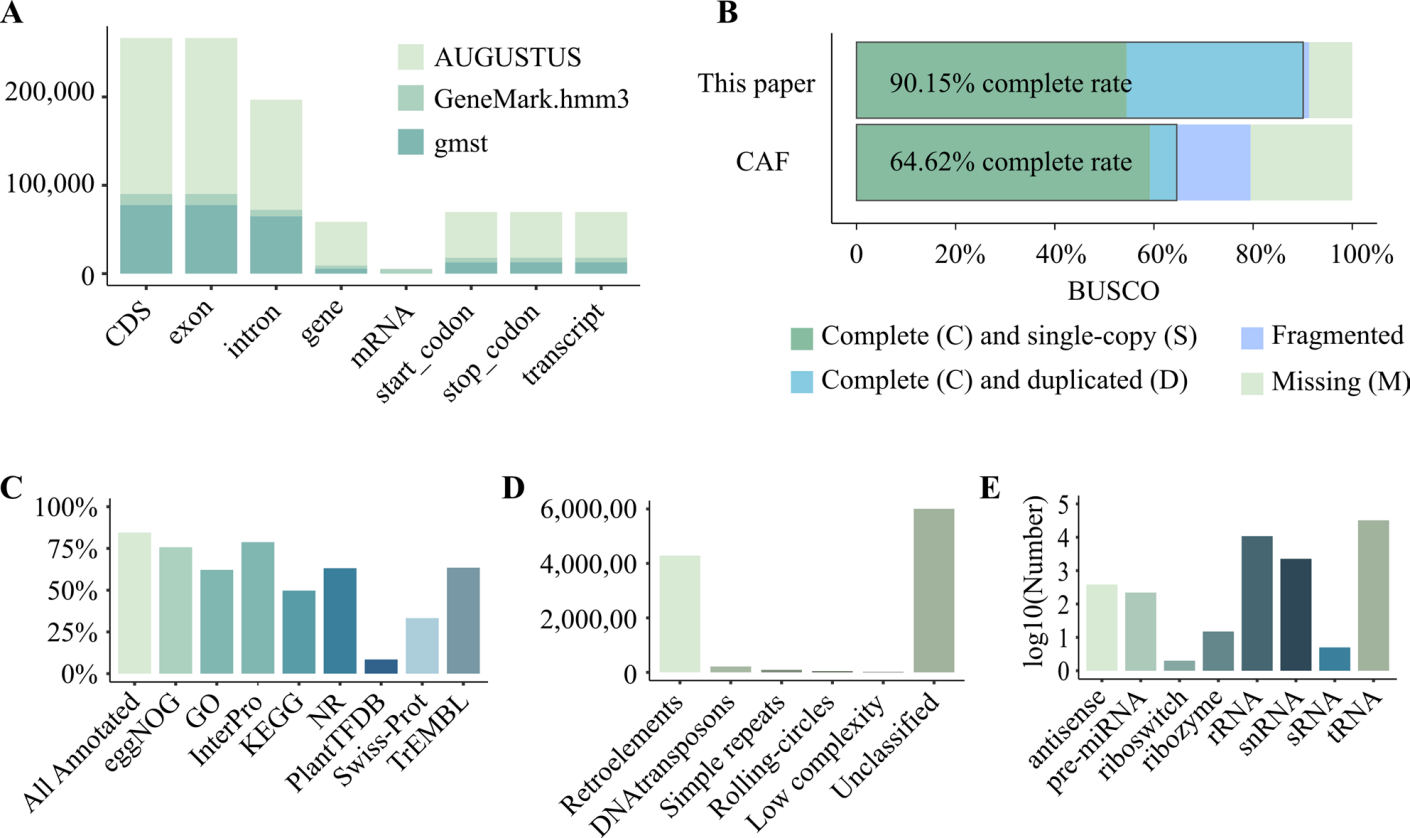
Structure and function annotations of *Larix kaempferi* genome. **A**: The number of features predicted in L. kaempferi genome. The y axis represents the number of features predicted in structure annotation. **B**: The comparison of BUSCO (The benchmarking sets of universal single-copy orthologs) assessment results between the structure annotation in the studies of this paper and Chinese Academy of Forestry (CAF) (Sun et al. 2022). The completeness of the structure annotation in this paper was 90.15%, much higher than that of CAF (64.62%). **C**: The percentage of annotated genes in each database. 84.5% of all genes were functionally annotated based on sequences similarity to at least one public database. **D**: The total length of recognized repetitive sequences. Length is presented in kbp. **E**: The number of annotated non-coding RNA. The number was transformed with log10 function

Genome structural annotation of *L. kaempferi* predicted five major genomic features using three complementary strategies: de novo prediction, protein homology, and transcriptome-based approaches. A total of 58,598 genes were annotated, along with 266,712 exons, 196,906 introns, 5,276 mRNAs, and 69,806 transcripts. Although the average intron length was 8,014 bp, the total intron length was the second longest among all annotated elements (Table 1; Table S4; Fig. 1A).

**Table 1:** The statistical results of genome structure annotation of *Larix Kaempferi*

Structure type	Number	Total length (bp)	Average length (bp)
exon	266,712	67,422,498	253
gene	58,598	1,138,916,600	19,436
intron	196,906	1,577,934,854	8,014
mRNA	5,276	10,003,232	1,896
transcript	69,806	1,645,357,352	23,570

Notes: Structural annotation was performed using BRAKER3 with 3 strategies: de novo prediction, protein homology, and transcriptome evidence. The number of each structure type isthe sum of the results from three prediction tools: AUGUSTUS, GeneMark.hmm3 and gmst

Assessment of annotation completeness using BUSCO (protein mode) revealed a high completeness rate of 90.15% (Table S5; Fig. 1B), exceeding that of the previous CAF genome version [23] and indicating a more accurate and comprehensive gene model set.

Of all annotated genes, 49,513 (84.5%) were functionally assigned based on sequence similarity to at least one public database. The highest annotation coverage was from InterPro (46,164 genes, 78.78%), while Swiss-Prot annotated the fewest genes (19,499 genes, 33.28%). Additionally, 4,973 (8.49%) genes were identified as transcription factors using the PlantTFDB database (Table 2; Fig. 1C).

**Table 2:** The statistical results of functional annotation of *Larix Kaempferi* genome

Public database	Number of annotated genes	Percentage
GO Annotation	36,430	62.17%
KEGG Annotation	29,145	49.74%
eggNOG Annotation	44,376	75.73%
NR Annotation	37,007	63.15%
InterPro Annotation	46,164	78.78%
Swiss-Prot Annotation	19,499	33.28%
TrEMBL Annotation	37,195	63.47%
PlantTFDB Annotation	4,973	8.49%
All Annotated Genes	49,513	84.50%

Notes: Functional annotation was implemented using the BLAST toolkit against seven databases (Swiss-Prot and TrEMBL belong to UniProt database) and the most similar sequence annotation was maintained

Repeat annotation showed that 78.96% (10.65 Gb) of the *L. kaempferi* genome consists of repetitive sequences. Among these sequences, retrotransposons accounted for 31.78%, including 28.31% long terminal repeat (LTR) elements and 14.79% Gypsy/DIRS1 elements. DNA transposons and tandem repeats made up 1.59% and 0.68%, respectively. Notably, 44.50% of repeats were unclassified, representing the single largest category (Table S6; Fig. 1D).

Non-coding RNA annotation identified 220 miRNA precursors (pre-miRNAs), 10,812 rRNAs, and 32,206 tRNAs, with average lengths of 117 bp, 1,162 bp, and 74 bp, respectively. Additionally, small nuclear RNAs (snRNAs) and antisense RNAs were also annotated, both showing substantial abundance (Table S7; Fig. 1E).

### Identification and functional enrichment of age-related genes

A total of 28.5 Gb of raw RNA-seq data were obtained from 16 samples. After quality control, 311,541,568 clean reads were retained, with mapping rates to the reference genome ranging from 89.70% to 94.78% (average 92.86%) (Table S8). Based on our novel genome annotation, 58,598 genes were used for downstream analysis. All RNA-seq datasets have been deposited in the CNCB GSA database (Table S8).

**Fig. 2:**
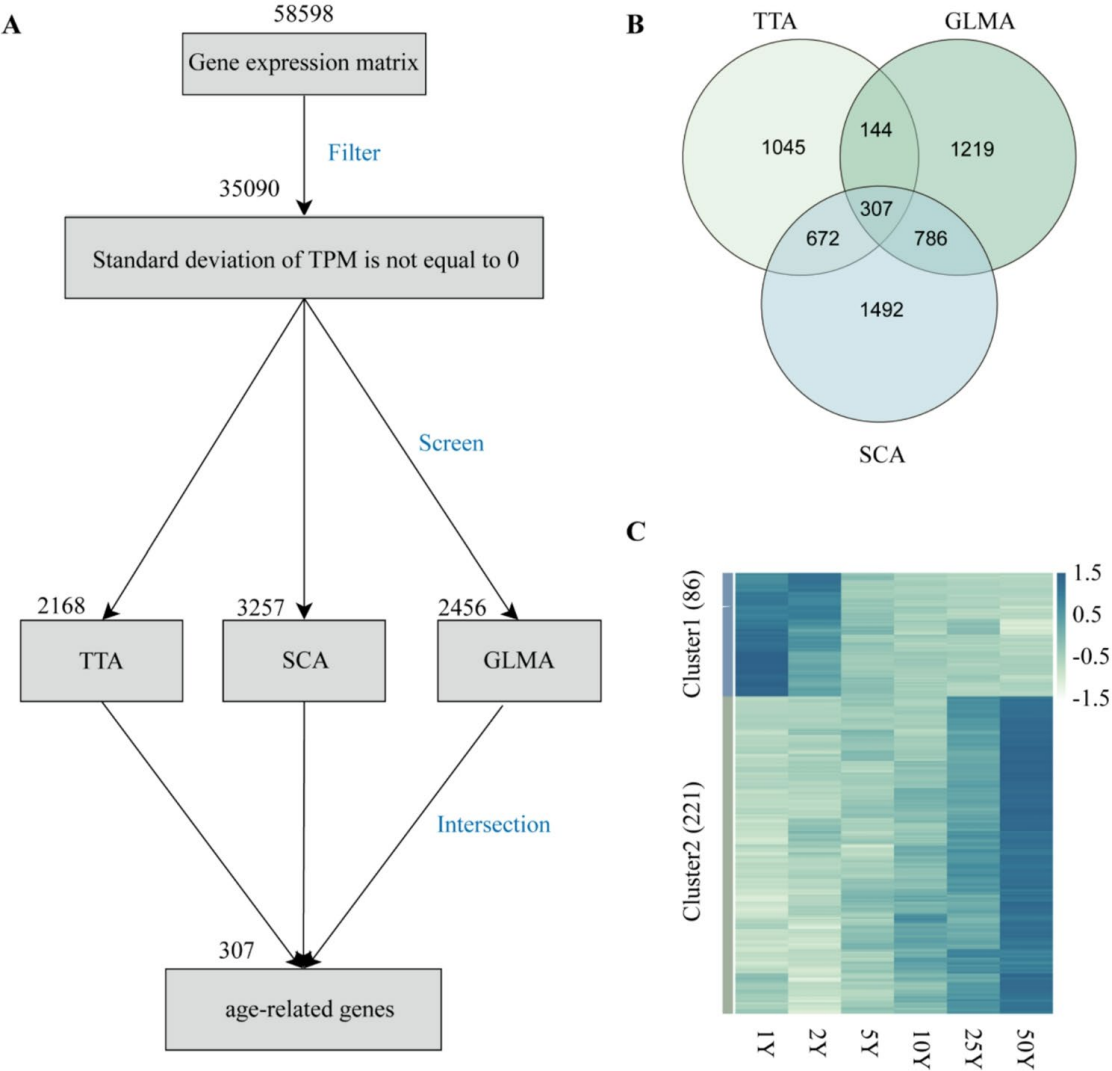
Identification and expression patterns of age-related genes (ARGs). **A**: Identification flowchart. 58598 genes were annotated from the BRAKER3 pipeline and then quantified in each sample. Among them, 35090 genes met our screen standards and then were analyzed to find genes that have a regular expression pattern over age using time trend analysis (TTA), spearman correlation analysis (SCA) and generalized linear model analysis (GLMA). Finally, 307 genes were identified as ARGs for further study. **B**: A Venn diagram to show the intersection of the results from TTA, SCA and GLMA. **C**: Expression patterns of 307 ARGs in age samples. The ARGs could be divided into two clusters, Cluster1 has a decreasing expression trend over age and Cluster2 has an opposing pattern

To identify age-related genes, we applied three independent methods. First, TTA identified 2,168 genes with consistent temporal expression patterns. Second, SCA identified 3,257 genes whose expression levels significantly correlated with sample age (Table S2). Third, GLMA identified 2,456 candidate age-related genes using empirical Bayes statistics. The intersection of these three methods yielded 307 high-confidence age-related genes (Fig. 2A-B).

These genes were clustered into two expression groups: Cluster1 (downregulated with age) and Cluster2 (upregulated with age) (Fig. 2C). Cluster2 contained 221 genes—more than twice the number in Cluster1 (Table S9).

**Fig. 3:**
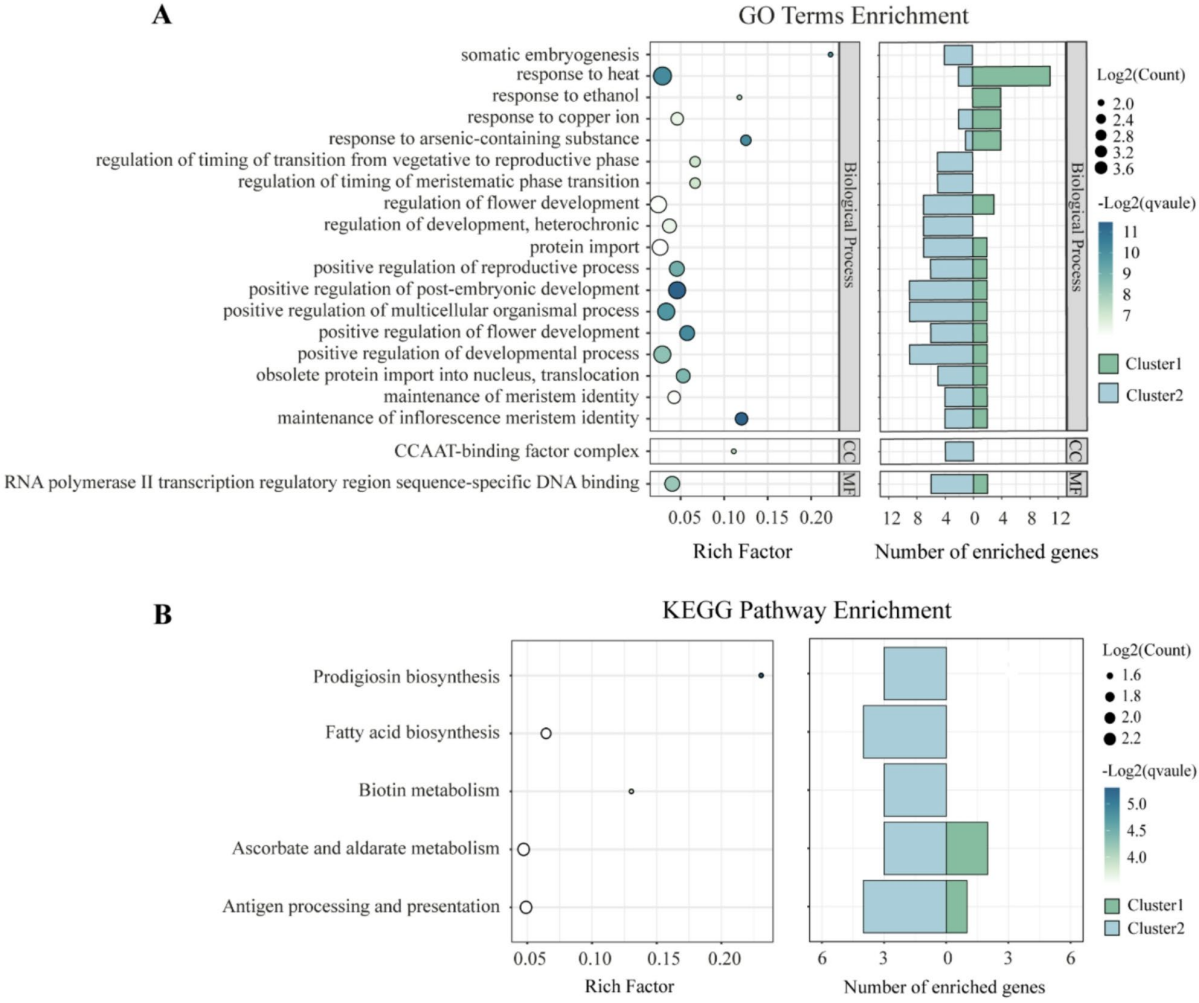
Gene Ontology (GO) terms and Kyoto Encyclopedia of Genes and Genomes (KEGG) pathways enrichment of age-related genes (ARGs). **A**: GO terms enrichment. Only head 20 terms with qvalue < 0.05 were shown here. MF, molecular function; CC, cellular component. The number of ARGs in each enriched GO term was shown here. **B**: KEGG pathway enrichment. Only pathways with qvalue < 0.2 were shown here. The number of ARGs in each enriched KEGG pathway was shown here

GO enrichment analysis was performed to explore biological functions. Among the 307 genes, 225 were annotated in the GO database. A total of 40 GO terms were significantly enriched, including 36 in the “biological process” category, three in “molecular function”, and one in “cellular component” (Table S10). Notably, the top three (according to the rich factor) enriched terms included “somatic embryogenesis,” “response to arsenic-containing substance,” and “maintenance of inflorescence meristem identity.” Cluster1 genes were predominantly enriched in abiotic stress-related terms, while Cluster2 genes were enriched in developmental functions (Fig. 3A).

KEGG pathway enrichment revealed five significantly associated pathways. Overall, age-related genes were enriched in “prodigiosin biosynthesis” and “biotin metabolism” (top two enriched pathways according to the rich factor). Cluster-specific enrichment showed that Cluster1 genes were uniquely associated with “ascorbate and aldarate metabolism” and “antigen processing and presentation” (Fig. 3B, Table S11).

### Spatiotemporal expression patterns of age-related genes

**Fig. 4:**
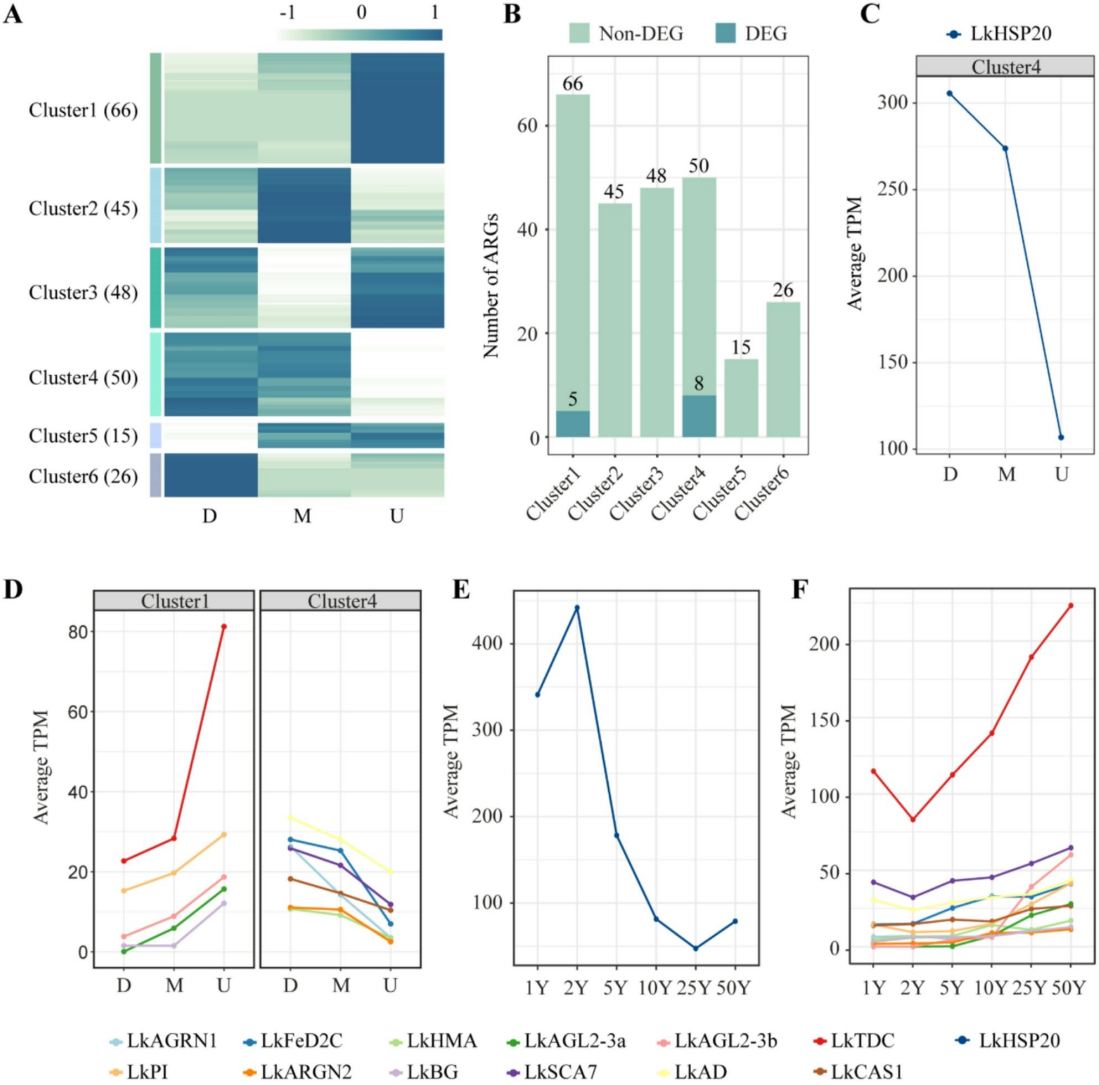
Spatial expression patterns of age-related genes (ARGs). **A**: Spatial expression patterns of ARGs. 250 fine genes were divided into six clusters, with various expression patterns in space. **B**: The number of ARGs in each cluster. Only Cluster1 and Cluster4 have ARGs that were differentially expressed in height gradient under a standard of pvalue < 0.05 and |logFC| > 0.5. Two comparison groups, D vs. M and M vs. U, were used to screen out differentially expressed genes (DEGs). **C**-**D**: The spatial expression patterns of 13 identified DEGs. Genes with an average TPM (Transcripts Per Million) higher than 100 were drawn alone. **E-F**: The temporal expression patterns of 13 identified DEGs. Genes with an average TPM higher than 100 were drawn alone

To investigate spatial variation in the expression of age-related genes, we analyzed RNA-seq data from height-gradient samples collected at three vertical positions—down (D), middle (M), and upper (U)—of 25-year-old *L. kaempferi* trees. After applying the expression stability filter (TPM = 0 across all samples or TPM SD > 50 within sample groups), 250 of the 307 age-related genes were retained for analysis. Hierarchical clustering using the ward.D2 method grouped these genes into six distinct clusters, designated Cluster1 through Cluster6. Cluster1 (genes highly expressed in the upper stem) contained the largest number of genes (66 genes), while Cluster5 (genes primarily expressed in the middle and upper stem) contained the fewest (15 genes) (Fig. 4A). 

To identify genes showing significant spatial expression differences, we performed differential expression analysis using edgeR for “D vs. M” and “M vs. U” pairwise comparisons. Only genes that were significantly differentially expressed in at least one comparison (*p* < 0.05, |log₂FC| > 0.5) were retained. This analysis identified 13 differentially expressed genes (DEGs), all belonging to Cluster1 or Cluster4 (Fig. 4B; Table 3).

**Table 3:** The basic information of differentially expressed age-related genes in height-gradient samples

Gene ID	Gene name	CAS	CHS	TF
g12361	*LkARGN1*	Cluster2	Cluster4	False
g15668	*LkFeD2C*	Cluster2	Cluster4	False
g20333	*LkHSP20*	Cluster1	Cluster4	False
g22031	*LkHMA*	Cluster2	Cluster4	False
g23306	*LaAGL2-3a*	Cluster2	Cluster1	MIKC_MADS
g23308	*LaAGL2-3b*	Cluster2	Cluster1	MIKC_MADS
g25649	*LkTDC*	Cluster2	Cluster1	False
g26505	*LkPI*	Cluster2	Cluster1	False
g26902	*LkARGN2*	Cluster2	Cluster4	False
g39379	*LkBG*	Cluster2	Cluster1	False
g39549	*LkSCA7*	Cluster2	Cluster4	False
g54894	*LkAD*	Cluster2	Cluster4	False
g55746	*LkCAS1*	Cluster2	Cluster4	False

Notes: CAS, the cluster id in Fig. 3B, Cluster1 demonstrated a decline in gene expression overage, whereas Cluster2 exhibited an increase. CHS, the cluster id in Fig. 5A. TF, the informationof transcription factors

Among these thirteen DEGs, two were annotated as MIKC-type MADS-box transcription factors (*LaAGL2-3a*, gene ID: g23306; *LaAGL2-3b*, gene ID: g23308), suggesting potential regulatory roles in spatial developmental processes. Notably, several genes displayed consistent temporal and spatial expression trends. For example, *LkHSP20* (gene ID: g20333), encoding a small heat shock protein, exhibited a high expression level in both young trees and lower stem regions (Fig. 4C, E), suggesting a role in early developmental stages. These 13 DEGs were used for further regulatory and functional characterization.

**Fig. 5:**
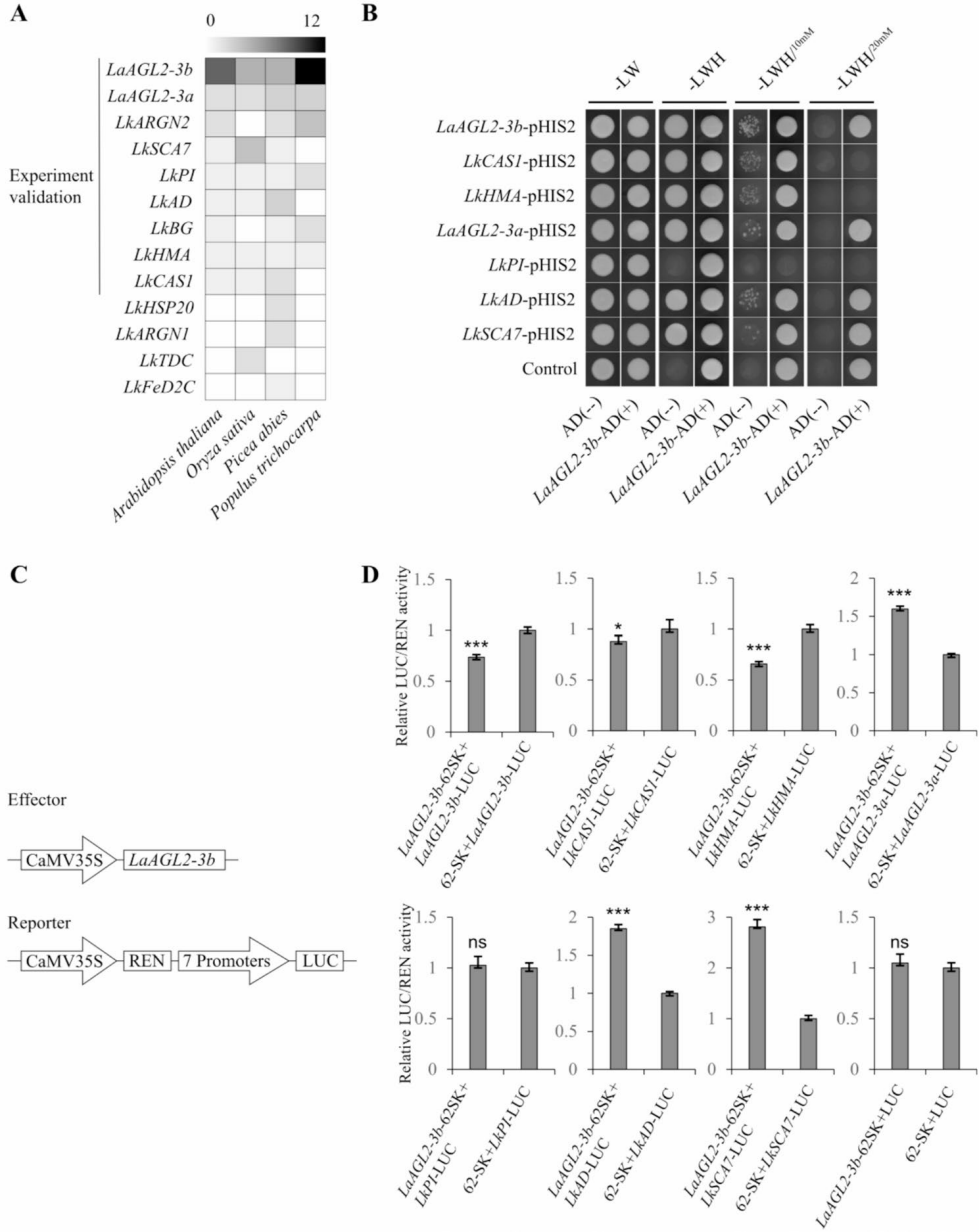
Analysis of interactions between *LaAGL2-3b* and other seven genes. **A**: Promoter binding site prediction. 13 age-related genes were submitted to predict potential transcription factor binding sites and the number of potential MADS-box transcription factor binding sites were presented here. We selected nine high confident genes (predicted to be bound in at least three species) for further experiment validation **B**: Yeast one-hybrid (Y1H) assay results. Y1H confirmed that L. kaempferi AGAMOUS*-Like (LaAGL2-3b)* directly binds to the promoters of seven genes: LaAGL2-3a, LaAGL2-3b (self-regulation), *L. kaempferi* *cycloartenol synthase (LkCAS1), LkHMA, LkPI, LkAD*, and *LkSCA7*. **C**: Schematic diagrams of the effector and reporter vector used in dual-luciferase reporter (DLR) assays. **D**: DLR assay results, which showed that LaAGL2-3b increased the promoter activity of *LaAGL2-3a*, LkAD and LkSCA7 and decreased the promoter activity of LaAGL2-3b (self-regulation), *LkCAS1 *and *LkHMA*. Values are the ratio of firefly luciferase (LUC) to Renilla luciferase (REN) activity. Data represents the mean of three biological replications. Error bars represent standard error. Statistical significance was determined using Student’s t-test. *** p ≤ 0.001, ** p ≤ 0.01, * p ≤ 0.05, ns p > 0.05

Regulatory role of *LaAGL2-3b* in age-related gene networks and developmental progression To explore potential regulatory relationships among these 13 differentially expressed age-related genes, promoter binding site analysis was performed. All 13 genes were predicted to contain MADS-box transcription factor binding sites (Fig. 5A), but only genes that were predicted to be bound in at least three species were selected for experiment validation. *LaAGL2-3a* and *LaAGL2-3b*, both MIKC-type MADS-box genes located on the same genomic scaffold, showed high similarity in primary structural and predicted tertiary structural levels (Figure S2). However, only *LaAGL2-3b* was successfully cloned.

Using full-length cDNA and promoter sequences, Y1H assays confirmed that *LaAGL2-3b* directly binds to the promoters of seven genes: *LaAGL2-3a*, *LaAGL2-3b* (self-regulation), *LkCAS1*, *LkHMA*, *LkPI*, *LkAD*, and *LkSCA7* (Fig. 5B). DLR assays further confirmed that LaAGL2-3b increased the promoter activity of *LaAGL2-3a*, *LkAD*, and *LkSCA7* and decreased the promoter activity of *LaAGL2-3b* (self-regulation), *LkCAS1*, and *LkHMA* (Fig. 5C-D). These results support a complex regulatory role for *LaAGL2-3b* within a spatially coordinated transcriptional network.

**Fig. 6:**
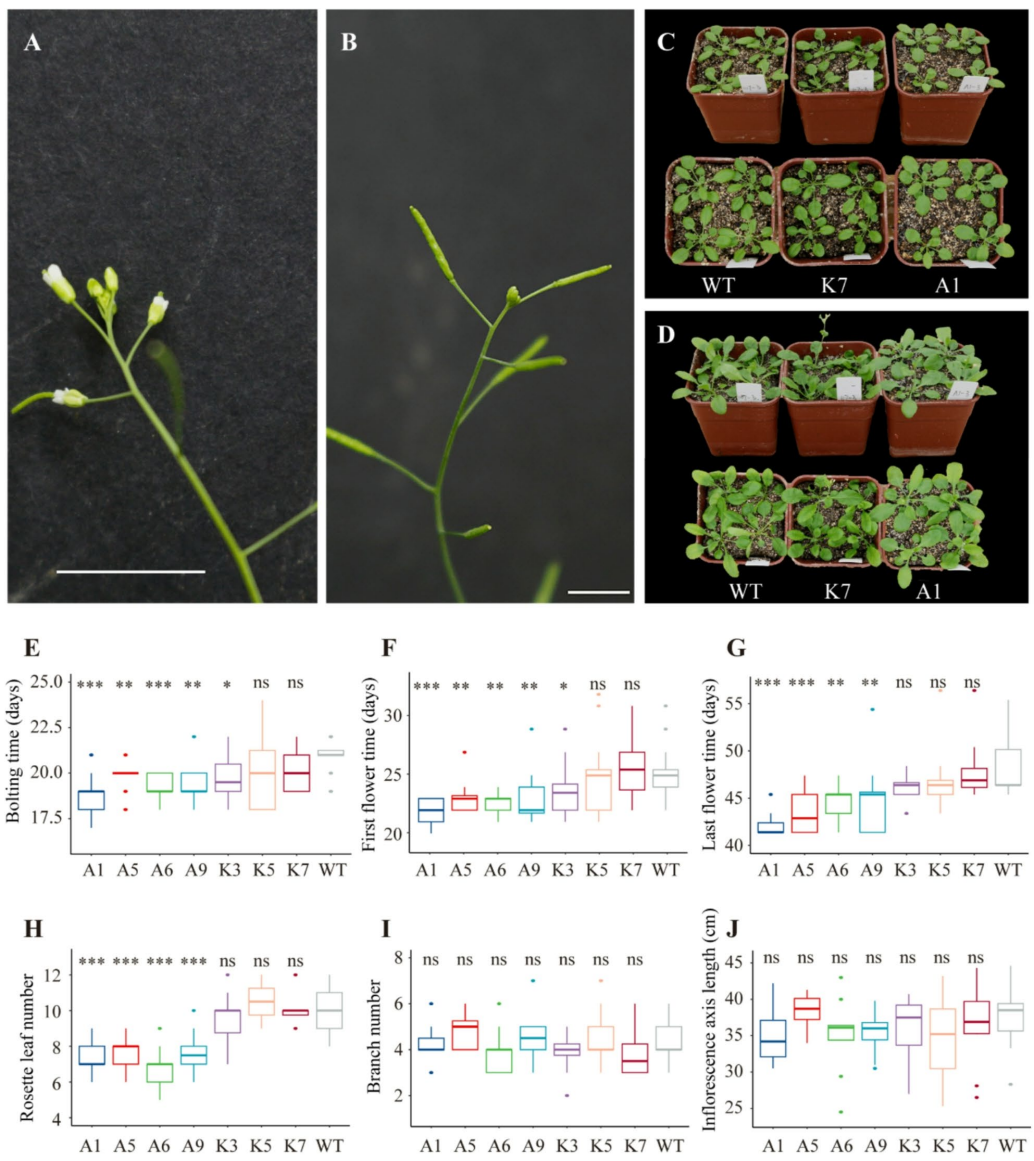
Phenotypic indices measured in wild-type (WT), negative control and positive control of *Arabidopsis thaliana*. Negative control is transgenic lines with plasmid empty vector (pK2FMCS7 empty, K3-K7). Positive control is transgenic lines with recombinant plasmid vector (pK2FMCS7 CaMV35S::LkAGL2-3, A1-A9). The Wilcoxon test was used to determine whether there is a significant difference between WT and other groups. *** p ≤ 0.001, ** p ≤ 0.01, * p ≤ 0.05, ns p > 0.05. At least 10 independent individuals in each line were measured. **A**: Image of the flower formed at the apex of the principal inflorescence of wild-type *A. thaliana* after 49 days when they were sown in soil. Bar 1 cm. **B**: Image of the spherical structure formed at the apex of the principal inflorescence of positive control *A. thaliana* after 49 days when they were sown in soil. Bar 1 cm. **C**: Image of wild-type (left), negative control (middle) and positive control (right) *A. thaliana* after 19 days when they were sown in soil. **D**: Image of wild-type (left), negative control (middle) and positive control (right) *A. thaliana* after 22 days when they were sown in soil. **E**: Bolting time of wild-type, negative control and positive control A. thaliana. **F**: First flower time of wild-type, negative control and positive control *A. thaliana*. **G**: Last flower time of wild-type, negative control and positive control *A. thaliana*. **H**: Rosette leaf number of wild-type, negative control and positive control *A. thaliana.*** I**: Branch number of wild-type, negative control and positive control *A. thaliana*. **J**: Inflorescence axis length of wild-type, negative control and positive control *A. thaliana.*

To assess the function of *LaAGL2-3* in plants, we over-expressed this gene in *A. thaliana* (Col-0). Transgenic lines exhibited significantly accelerated developmental transitions compared to wild-type lines (Fig. 6A-D). On average, bolting occurred 2 days earlier, first flowering 2.8 days earlier, and final flower formation 5 days earlier; in addition, 2.53 fewer rosette leaves were produced in over-expression lines. However, no differences in the phenotypic indices of branch number and inflorescence axis length were found between over-expression and wild-type lines (Fig. 6E-J).

Altogether, these findings demonstrate that *LaAGL2-3b* can bind the promoters of several candidate genes and regulate their expressions. Its over-expression in *A. thaliana* alters developmental timing, suggesting a potential involvement in age-associated transcriptional and biological processes.

## Discussion

The availability of a high-quality reference genome and the integration of three complementary analytical approaches in the present study allow us to extend the previous findings [7, 19, 20, 22]. Earlier studies identified age-associated transcription factors mainly through transcriptome assemblies, whereas our genome-guided workflow enables more accurate mapping of reads and improves coding gene discovery and the detection of age-related genes beyond transcription factors. By combining TTA, SCA, and GLMA, rather than relying solely on the analysis of differentially expressed genes, we substantially increased the robustness and confidence of age-related gene identification, providing a more comprehensive and refined view of the molecular landscape underlying *L. kaempferi* aging.

### Genome structure annotation of *L. kaempferi* facilitates the study of biological function and evolution

The availability of a high-quality reference genome has revolutionized molecular biology and genetics, enabling more accurate gene discovery, evolutionary inference, and functional studies. However, the utility of genomic data critically depends on the accuracy and completeness of its annotation, as erroneous or incomplete annotations can mislead downstream analysis and compromise biological interpretation [55–57].

In this study, we presented an updated structural annotation of the *L. kaempferi* genome based on the most recent assembly, which demonstrates substantial improvements over the previous CAF version in terms of assembly continuity (reduced contig number and increased contig N50; Table S1). By integrating both publicly available protein datasets and our own large-scale transcriptomic resources, we annotated a higher number of high-confidence protein-coding genes (Table S4). Additionally, we identified a higher proportion of repetitive elements (Table S6), a feature more comparable to the genome of *Pinus lambertiana* Douglas [58]. Importantly, the completeness of our annotation was validated using BUSCO analysis, which revealed a higher percentage of complete BUSCOs (both single-copy and duplicated) compared to previous versions (Table S5, Fig. 1B). Notably, the elevated rate of duplicated BUSCOs should be interpreted with caution, because gymnosperm genomes typically contain many large gene families that expanded in ancient times, and genuine paralogous copies are frequently under-represented or collapsed in fragmented assemblies [59, 60]. In addition, some duplicated BUSCOs may also result from incomplete removal of alternative haplotypes, a known challenge in assembling large and highly heterozygous conifer genomes [61].

Together, these improvements provide a more accurate and comprehensive genomic framework for *L. kaempferi*, which will facilitate downstream investigations of gene function, regulatory mechanisms, and evolutionary dynamics in this ecologically and economically important conifer species.

### Temporal transcriptomic profiling reveals age-dependent shifts in stress responses and developmental program in *L. kaempferi*

Tree aging is accompanied by dynamic changes in physiological traits such as abiotic stress responses and reproductive development [62, 63]. Understanding the molecular mechanisms underlying these transitions is essential for improving our knowledge of long-term plant adaptation and informing forest management strategies. In the present study, we analyzed transcriptomic data across an age gradient sample of *L. kaempferi* using three complementary statistical approaches (Fig. 2B) and identified a set of age-related genes. Functional enrichment analysis suggests that two major expression clusters may represent different biological emphases during tree aging. Genes in Cluster1 (Fig. 2C), which show decreasing expression across the age gradient, are enriched for stress-related GO terms. This pattern implies that stress-responsive processes could be more transcriptionally prominent during the early stages of growth, when young trees experience rapid establishment and may be more sensitive to environmental fluctuations. Conversely, genes in Cluster2 (Fig. 2C), which show increasing expression across the age gradient, are enriched in developmental and biosynthetic categories. Such enrichment is consistent with the notion that, as trees mature, processes related to tissue differentiation, secondary growth, and biomass accumulation gradually become more transcriptionally represented (Fig. 3).

Among the identified age-related genes, two stand out as representative markers of age-dependent physiological transitions: *LkHSP20* and *LkTDC*. *LkHSP20* encodes a small heat shock protein, a class of molecular chaperones that help maintain protein homeostasis under stress by refolding denatured proteins [64, 65]. This gene exhibits high expression levels in young trees followed by a consistent decline with age (Fig. 4C, E), a pattern consistent with previous observations in *Pinus koraiensis* Siebold & Zucc., where 15 heat shock protein genes show peak expression at age 5 and then decrease with age [66]. In contrast, *LkTDC*, involved in the jasmonic acid signaling pathway and known to function as a suppressor in the upstream of floral organ identity regulatory network in *O. sativa* [67], shows a similar age-dependent expression with an increasing pattern during tree aging (Fig. 4D, F). Together, these results demonstrate that traits like stress response and reproduction change with age in *L. kaempferi*, and it is possible to capture these dynamics with gene expression. Future studies focusing on the upstream regulatory pathway and transcriptional regulatory networks of these genes will deepen our understanding of age-mediated stress adaptation and development in conifers.

### Spatiotemporal expression and regulatory network of age-related genes reveal the molecular basis of phenotypic and physiological transitions in *L. kaempferi*

*AGAMOUS-like 6* (*AGL6*) genes encode MIKC-type MADS box transcription factors that have been primarily studied in angiosperms for their roles in floral meristem identity [68]. In *A. thaliana* and *O. sativa*,* AGL6* genes function redundantly with other floral homeotic regulators to control meristem fate during flower development [68–71]. In the present study, the *L. kaempferi AGL6* homolog, *LaAGL2-3b*, was identified as an age-related gene through spatiotemporal transcriptomic analysis. Its over-expression in *A. thaliana* resulted in a significant acceleration in life cycle progression (Fig. 6), supporting the hypothesis that *LaAGL2-3b* may share conserved functional features with angiosperm *AGL6-like* genes.

In addition to *LaAGL2-3b*, we identified two other functionally relevant genes involved in hormonal signaling: *LkTDC*, associated with the jasmonic acid pathway and known to be a suppressor in the upstream of the regulatory network of *AGL6-like* genes in floral development [67, 72–74], and *LkPI*, encoding a peptidyl-prolyl cis-trans isomerase that regulates auxin transport via modulation of ABC transporters [75]. These genes exhibited coordinated age-dependent expression patterns both temporally and spatially (Fig. 4D, F), suggesting that these genes may serve as candidates for exploring molecular signatures of ontogenetic age.

Age-related regulation also extends to traits associated with wood formation. In conifers, xylem development involves the cyclical production of earlywood and latewood, with wood density being a key mechanical trait dependent on their relative proportions [76]. We identified two age-related genes linked to this process: *LkHMA*, encoding a heavy-metal transporter potentially involved in lignin biosynthesis via copper homeostasis [77–79], and *LkFeD2C*, a 2-oxoglutarate and Fe(II)-dependent dioxygenase that likely regulates secondary cell wall formation through gibberellin-mediated suppression of DELLA proteins and activation of NAC/MYB transcription factors [80, 81]. These observations raise the possibility that age-dependent transcriptional changes may be associated with vertical variation in wood-related physiological processes.

Strikingly, our Y1H and DLR assays demonstrate that LaAGL2-3b directly binds to the promoters of six age-related genes, including *LaAGL2-3a*, *LaAGL2-3b*, *LkCAS1*, *LkHMA*, *LkAD*, and *LkSCA7* (Fig. 5), all of which show spatial differential expression patterns (Fig. 4C-F). This suggests that *LaAGL2-3* may participate in integrating temporal and spatial expression patterns within age-associated transcriptional networks.

Taken together, our results uncover a regulatory framework in which age-related transcription factors *LaAGL2-3* may be positioned upstream of pathways associated with development and wood formation, although the precise regulatory mechanisms remain to be investigated. These insights bridge the gap between molecular aging and observable phenotypes in trees and lay the groundwork for future research on genetic modulation of aging traits in long-lived woody species.

## Conclusions

In this study, we generated a new structural annotation of the *L. kaempferi* genome and integrated it with spatiotemporal transcriptomic analysis to investigate age-dependent developmental variation in *L. kaempferi*. We identified 307 robust age-related genes, among which 13 showed clear vertical expression gradients within the stem, suggesting their coordinated age-associated transcription patterns across spatial positions. We further showed that *LaAGL2-3b*, a MIKC-type MADS-box transcription factor, directly binds to the promoters of several of these 13 genes, including *LaAGL2-3a*, *LaAGL2-3b*, *LkCAS1*, *LkHMA*, *LkAD*, and *LkSCA7*, and regulates their expression. Over-expression of *LaAGL2-3* in *A. thaliana* altered developmental timing, supporting a potential involvement of this gene in developmental regulatory processes. Collectively, these findings provide a set of age-related genes and functional hypotheses for understanding transcriptional programs associated with aging in *L. kaempferi*, along with valuable genomic and transcriptomic resources for future functional and developmental studies in conifers.

## Supplementary Information

Supplementary Material 1: Figure S1-S2.

Supplementary Material 2: Table S1-S11.

Supplementary Material 3: The structure annotation of *Larix kaempferi *genome.

## Data Availability

All RNA-Seq data that supports this study have been deposited in the CNCB GSA database under accession number PRJCA030694. The structure annotation of the genome, table s1-s11, and figure s1-s2 are available as Supplementary Information at *BMC Plant Biology Online* .

## References

[1] Jiang Y, Zhang X, Chhin S, Zhang J. A bimodal pattern and age-related growth of intra-annual wood cell development of Chinese Fir in subtropical China. Front Plant Sci. 2021;12:757438. 10.3389/fpls.2021.757438. 34956260 10.3389/fpls.2021.757438 PMC8695768

[2] Liao X, Su Y, Klintenas M, Li Y, Sane S, Wu Z, Chen Q, Zhang B, Nilsson O, Ding J. Age-dependent seasonal growth cessation in *Populus*. Proc Natl Acad Sci U S A. 2023;120(48):e2311226120. 10.1073/pnas.2311226120. 37991940 10.1073/pnas.2311226120 PMC10691234

[3] Cato S, McMillan L, Donaldson L, Richardson T, Echt C, Gardner R. Wood formation from the base to the crown in *Pinus radiata*: gradients of tracheid wall thickness, wood density, radial growth rate and gene expression. Plant Mol Biol. 2006;60(4):565–81. 10.1007/s11103-005-5022-9. 16525892 10.1007/s11103-005-5022-9

[4] England JR, Attiwill PM. Changes in leaf morphology and anatomy with tree age and height in the broadleaved evergreen species, *Eucalyptus regnans* F. Muell. Trees. 2005;20(1):79. 10.1007/s00468-005-0015-5. 10.1093/treephys/27.8.1113 17472938

[5] Paiva JAP, Garces M, Alves A, Garnier-Gere P, Rodrigues JC, Lalanne C, Porcon S, Le Provost G, Da Silva Perez D, Brach J, et al. Molecular and phenotypic profiling from the base to the crown in maritime pine wood-forming tissue. New Phytol. 2008;178(2):283–301. 10.1111/j.1469-8137.2008.02379.x. 18298434 10.1111/j.1469-8137.2008.02379.x

[6] Garces M, Le Provost G, Lalanne C, Claverol S, Barre A, Plomion C, Herrera R. Proteomic analysis during ontogenesis of secondary xylem in maritime pine. Tree Physiol. 2014;34(11):1263–77. 10.1093/treephys/tpt117. 24614303 10.1093/treephys/tpt117

[7] Zhang Y, Zang Q-L, Qi L-W, Han S-Y, Li W-F. Effects of cutting, pruning, and grafting on the expression of age-related genes in *Larix Kaempferi*. Forests. 2020;11(2):218. 10.3390/f11020218.

[8] Jung JH, Ju Y, Seo PJ, Lee JH, Park CM. The SOC1-SPL module integrates photoperiod and gibberellic acid signals to control flowering time in *Arabidopsis*. Plant J. 2012;69(4):577–88. 10.1111/j.1365-313X.2011.04813.x. 21988498 10.1111/j.1365-313X.2011.04813.x

[9] Murai K, Miyamae M, Kato H, Takumi S, Ogihara Y. *WAP1*, a wheat *APETALA1* homolog, plays a central role in the phase transition from vegetative to reproductive growth. Plant Cell Physiol. 2003;44(12):1255–65. 10.1093/pcp/pcg171. 14701921 10.1093/pcp/pcg171

[10] Preston JC, Hileman LC. Functional evolution in the plant *SQUAMOSA-PROMOTER BINDING PROTEIN-LIKE* (*SPL*) gene family. Front Plant Sci. 2013;4:80. 10.3389/fpls.2013.00080. 23577017 10.3389/fpls.2013.00080 PMC3617394

[11] Yu N, Yang JC, Yin GT, Li RS, Zou WT. Genome-wide characterization of the SPL gene family involved in the age development of *Jatropha Curcas*. BMC Genomics. 2020;21(1):368. 10.1186/s12864-020-06776-8. 32434522 10.1186/s12864-020-06776-8 PMC7238634

[12] Carlsbecker A, Tandre K, Johanson U, Englund M, Engstrom P. The MADS-box gene *DAL1* is a potential mediator of the juvenile-to-adult transition in Norway Spruce (*Picea abies*). Plant J. 2004;40(4):546–57. 10.1111/j.1365-313X.2004.02226.x. 15500470 10.1111/j.1365-313X.2004.02226.x

[13] Wang JW, Park MY, Wang LJ, Koo Y, Chen XY, Weigel D, Poethig RS. MiRNA control of vegetative phase change in trees. PLoS Genet. 2011;7(2):e1002012. 10.1371/journal.pgen.1002012. 21383862 10.1371/journal.pgen.1002012 PMC3044678

[14] Curaba J, Talbot M, Li Z, Helliwell C. Over-expression of microRNA171 affects phase transitions and floral meristem determinancy in barley. BMC Plant Biol. 2013;13(1):6. 10.1186/1471-2229-13-6. 23294862 10.1186/1471-2229-13-6 PMC3547705

[15] Mimida N, Kotoda N, Ueda T, Igarashi M, Hatsuyama Y, Iwanami H, Moriya S, Abe K. Four *TFL1/CEN*-like genes on distinct linkage groups show different expression patterns to regulate vegetative and reproductive development in Apple (*Malus* x *domestica* Borkh). Plant Cell Physiol. 2009;50(2):394–412. 10.1093/pcp/pcp001. 19168455 10.1093/pcp/pcp001

[16] Fernández-Ocaña A, Carmen García-López M, Jiménez-Ruiz J, Saniger L, Macías D, Navarro F, Oya R, Belaj A, de la Rosa R, Corpas FJ, et al. Identification of a gene involved in the juvenile-to-adult transition (JAT) in cultivated Olive trees. Tree Genet Genomes. 2010;6(6):891–903. 10.1007/s11295-010-0299-5.

[17] Li A, Zhou Y, Jin C, Song W, Chen C, Wang C. *LaAP2L1*, a heterosis-associated AP2/EREBP transcription factor of *Larix*, increases organ size and final biomass by affecting cell proliferation in *Arabidopsis*. Plant Cell Physiol. 2013;54(11):1822–36. 10.1093/pcp/pct124. 24009335 10.1093/pcp/pct124

[18] Gao J, Zhang K, Cheng YJ, Yu S, Shang GD, Wang FX, Wu LY, Xu ZG, Mai YX, Zhao XY, et al. A robust mechanism for resetting juvenility during each generation in *Arabidopsis*. Nat Plants. 2022;8(3):257–68. 10.1038/s41477-022-01110-4. 35318444 10.1038/s41477-022-01110-4

[19] Ye Z-L, Zang Q-L, Cheng D-X, Li X-Y, Qi L-W, Li W-F. Over-expression of larch *DAL1* accelerates life-cycle progression in *Arabidopsis*. Forests. 2022;13(6):953. 10.3390/f13060953.

[20] Li XY, Ye ZL, Cheng DX, Zang QL, Qi LW, Li WF. *LaDAL1* coordinates age and environmental signals in the life cycle of *Larix Kaempferi*. Int J Mol Sci. 2022;24(1):426. 10.3390/ijms24010426. 36613870 10.3390/ijms24010426 PMC9820328

[21] Yang S, Zhang G, Zhang X, Lin C, Huang T, Deng L, Zhang Z, Li F, Zhong S, Pan X, et al. The ontogenetic ageing pattern and the molecular mechanism for Prunning rejuvenation in *Pinus Elliottii* × *P. caribaea*. SCI SIN Vitae. 2023;53(8):1146–65. 10.1360/ssv-2022-0259.

[22] Xiang W-B, Li W-F, Zhang S-G, Qi L-W. Transcriptome-wide analysis to dissect the transcription factors orchestrating the phase change from vegetative to reproductive development in *Larix Kaempferi*. Tree Genet Genomes. 2019;15(5):1–9. 10.1007/s11295-019-1376-z.30546292

[23] Sun C, Xie YH, Li Z, Liu YJ, Sun XM, Li JJ, Quan WP, Zeng QY, Van de Peer Y, Zhang SG. The *Larix Kaempferi* genome reveals new insights into wood properties. J Integr Plant Biol. 2022;64(7):1364–73. 10.1111/jipb.13265. 35442564 10.1111/jipb.13265

[24] Shirasawa K, Mishima K, Hirakawa H, Hirao T, Tsubomura M, Nagano S, Iki T, Isobe S, Takahashi M. Haplotype-resolved *de Novo* genome assemblies of four coniferous tree species. J for Res. 2023;29(2):151–7. 10.1080/13416979.2023.2267304.

[25] Li W-F, Yang W-H, Zhang S-G, Han S-Y, Qi L-W. Transcriptome analysis provides insights into wood formation during larch tree aging. Tree Genet Genomes. 2017;13(1):19. 10.1007/s11295-017-1106-3.

[26] Li W, Han S, Qi L, Zhang S. Transcriptome resources and genome-wide marker development for Japanese larch (*Larix kaempferi*). Front Agric Sci Eng. 2014;1(1):77–84. 10.15302/j-fase-2014010.

[27] Benson G. Tandem repeats finder: a program to analyze DNA sequences. Nucleic Acids Res. 1999;27(2):573–80. 10.1093/nar/27.2.573. 9862982 10.1093/nar/27.2.573 PMC148217

[28] Bao Z, Eddy SR. Automated *de Novo* identification of repeat sequence families in sequenced genomes. Genome Res. 2002;12(8):1269–76. 10.1101/gr.88502. 12176934 10.1101/gr.88502 PMC186642

[29] Price AL, Jones NC, Pevzner PA. *De novo* identification of repeat families in large genomes. *Bioinformatics* 2005, 21 Suppl 1(suppl_1):i351–358 10.1093/bioinformatics/bti1018 10.1093/bioinformatics/bti1018 15961478

[30] Hoff KJ, Lange S, Lomsadze A, Borodovsky M, Stanke M. BRAKER1: unsupervised RNA-seq-based genome annotation with GeneMark-ET and AUGUSTUS. Bioinformatics. 2016;32(5):767–9. 10.1093/bioinformatics/btv661. 26559507 10.1093/bioinformatics/btv661 PMC6078167

[31] Hoff KJ, Lomsadze A, Borodovsky M, Stanke M. Whole-Genome annotation with BRAKER. Methods Mol Biol. 2019;1962:65–95. 10.1007/978-1-4939-9173-0_5. 31020555 10.1007/978-1-4939-9173-0_5 PMC6635606

[32] Bruna T, Hoff KJ, Lomsadze A, Stanke M, Borodovsky M. BRAKER2: automatic eukaryotic genome annotation with GeneMark-EP + and AUGUSTUS supported by a protein database. NAR Genom Bioinform. 2021;3(1):lqaa108. 10.1093/nargab/lqaa108. 33575650 10.1093/nargab/lqaa108 PMC7787252

[33] Gabriel L, Bruna T, Hoff KJ, Ebel M, Lomsadze A, Borodovsky M, Stanke M. BRAKER3: fully automated genome annotation using RNA-seq and protein evidence with GeneMark-ETP, AUGUSTUS and TSEBRA. *BioRxiv* 2024:2023.2026.2010.544449 10.1101/2023.06.10.544449 10.1101/gr.278090.123 PMC11216308 38866550

[34] Mishima K, Hirakawa H, Iki T, Fukuda Y, Hirao T, Tamura A, Takahashi M. Comprehensive collection of genes and comparative analysis of full-length transcriptome sequences from Japanese larch (*Larix kaempferi*) and Kuril larch (*Larix Gmelinii* var. *japonica*). BMC Plant Biol. 2022;22(1):470. 10.1186/s12870-022-03862-9. 36192701 10.1186/s12870-022-03862-9 PMC9531402

[35] Kim D, Paggi JM, Park C, Bennett C, Salzberg SL. Graph-based genome alignment and genotyping with HISAT2 and HISAT-genotype. Nat Biotechnol. 2019;37(8):907–15. 10.1038/s41587-019-0201-4. 31375807 10.1038/s41587-019-0201-4 PMC7605509

[36] Kovaka S, Zimin AV, Pertea GM, Razaghi R, Salzberg SL, Pertea M. Transcriptome assembly from long-read RNA-seq alignments with StringTie2. Genome Biol. 2019;20(1):278. 10.1186/s13059-019-1910-1. 31842956 10.1186/s13059-019-1910-1 PMC6912988

[37] Tang S, Lomsadze A, Borodovsky M. Identification of protein coding regions in RNA transcripts. Nucleic Acids Res. 2015;43(12):e78. 10.1093/nar/gkv227. 25870408 10.1093/nar/gkv227 PMC4499116

[38] Stanke M, Schoffmann O, Morgenstern B, Waack S. Gene prediction in eukaryotes with a generalized hidden Markov model that uses hints from external sources. BMC Bioinf. 2006;7(1):62. 10.1186/1471-2105-7-62. 10.1186/1471-2105-7-62 PMC1409804 16469098

[39] Stanke M, Diekhans M, Baertsch R, Haussler D. Using native and syntenically mapped cDNA alignments to improve *de Novo* gene finding. Bioinformatics. 2008;24(5):637–44. 10.1093/bioinformatics/btn013. 18218656 10.1093/bioinformatics/btn013

[40] Bruna T, Lomsadze A, Borodovsky M. A new gene finding tool GeneMark-ETP significantly improves the accuracy of automatic annotation of large eukaryotic genomes. BioRxiv. 2024;2023(2021):2013–524024. 10.1101/2023.01.13.524024. 10.1101/gr.278373.123 PMC11216313 38866548

[41] Gabriel L, Hoff KJ, Bruna T, Borodovsky M, Stanke M. TSEBRA: transcript selector for BRAKER. BMC Bioinf. 2021;22(1):566. 10.1186/s12859-021-04482-0. 10.1186/s12859-021-04482-0 PMC8620231 34823473

[42] Manni M, Berkeley MR, Seppey M, Simao FA, Zdobnov EM. BUSCO update: novel and streamlined workflows along with broader and deeper phylogenetic coverage for scoring of eukaryotic, prokaryotic, and viral genomes. Mol Biol Evol. 2021;38(10):4647–54. 10.1093/molbev/msab199. 34320186 10.1093/molbev/msab199 PMC8476166

[43] Nawrocki EP, Eddy SR. Infernal 1.1: 100-fold faster RNA homology searches. Bioinformatics. 2013;29(22):2933–5. 10.1093/bioinformatics/btt509. 24008419 10.1093/bioinformatics/btt509 PMC3810854

[44] Kalvari I, Nawrocki EP, Ontiveros-Palacios N, Argasinska J, Lamkiewicz K, Marz M, Griffiths-Jones S, Toffano-Nioche C, Gautheret D, Weinberg Z, et al. Rfam 14: expanded coverage of metagenomic, viral and MicroRNA families. Nucleic Acids Res. 2021;49(D1):D192–200. 10.1093/nar/gkaa1047. 33211869 10.1093/nar/gkaa1047 PMC7779021

[45] Chen S, Zhou Y, Chen Y, Gu J. Fastp: an ultra-fast all-in-one FASTQ preprocessor. Bioinformatics. 2018;34(17):i884–90. 10.1093/bioinformatics/bty560. 30423086 10.1093/bioinformatics/bty560 PMC6129281

[46] Danecek P, Bonfield JK, Liddle J, Marshall J, Ohan V, Pollard MO, Whitwham A, Keane T, McCarthy SA, Davies RM et al. Twelve years of SAMtools and BCFtools. *GigaScience* 2021, 10(2)10.1093/gigascience/giab008 10.1093/gigascience/giab008 PMC7931819 33590861

[47] Liao Y, Smyth GK, Shi W. FeatureCounts: an efficient general purpose program for assigning sequence reads to genomic features. Bioinformatics. 2014;30(7):923–30. 10.1093/bioinformatics/btt656. 24227677 10.1093/bioinformatics/btt656

[48] Mölder F, Jablonski KP, Letcher B, Hall MB, Tomkins-Tinch CH, Sochat V, Forster J, Lee S, Twardziok SO, Kanitz A et al. Sustainable data analysis with Snakemake. In.: F1000Research; 202110.12688/f1000research.29032.1 10.12688/f1000research.29032.1 PMC8114187 34035898

[49] Liao T, Li W. Snakemake workflow: rna-seq-std. : Zenodo. 2025. 10.5281/zenodo.15148691.

[50] Wu T, Hu E, Xu S, Chen M, Guo P, Dai Z, Feng T, Zhou L, Tang W, Zhan L, et al. ClusterProfiler 4.0: A universal enrichment tool for interpreting omics data. Innov (Camb). 2021;2(3):100141. 10.1016/j.xinn.2021.100141. 10.1016/j.xinn.2021.100141 PMC8454663 34557778

[51] Robinson MD, McCarthy DJ, Smyth GK. EdgeR: a bioconductor package for differential expression analysis of digital gene expression data. Bioinformatics. 2010;26(1):139–40. 10.1093/bioinformatics/btp616. 19910308 10.1093/bioinformatics/btp616 PMC2796818

[52] Jin J, Tian F, Yang DC, Meng YQ, Kong L, Luo J, Gao G. PlantTFDB 4.0: toward a central hub for transcription factors and regulatory interactions in plants. Nucleic Acids Res. 2017;45(D1):D1040–5. 10.1093/nar/gkw982. 27924042 10.1093/nar/gkw982 PMC5210657

[53] Zhao H, Liao H, Li S, Zhang R, Dai J, Ma P, Wang T, Wang M, Yuan Y, Fu X, et al. Delphinieae flowers originated from the rewiring of interactions between duplicated and diversified floral organ identity and symmetry genes. Plant Cell. 2023;35(3):994–1012. 10.1093/plcell/koac368. 36560915 10.1093/plcell/koac368 PMC10015166

[54] Xing J, Zhang Q, Ye Z, Zhang C, Cheng D, Qi L, Yang L, Li W. Optimization and application of transient transformation system of *Larix Kaempferi*. For Res. 2024;37(03):129–35. 10.12403/j.1001-1498.20230390.

[55] Yandell M, Ence D. A beginner’s guide to eukaryotic genome annotation. Nat Rev Genet. 2012;13(5):329–42. 10.1038/nrg3174. 22510764 10.1038/nrg3174

[56] Armstrong J, Fiddes IT, Diekhans M, Paten B. Whole-genome alignment and comparative annotation. Annu Rev Anim Biosci. 2019;7:41–64. 10.1146/annurev-animal-020518-115005. 30379572 10.1146/annurev-animal-020518-115005 PMC6450745

[57] Rice ES, Green RE. New approaches for genome assembly and scaffolding. *Annu Rev Anim Biosci* 2019, 7(Volume 7, 2019):17–40 10.1146/annurev-animal-020518-115344 10.1146/annurev-animal-020518-115344 30485757

[58] Stevens KA, Wegrzyn JL, Zimin A, Puiu D, Crepeau M, Cardeno C, Paul R, Gonzalez-Ibeas D, Koriabine M, Holtz-Morris AE, et al. Sequence of the sugar pine megagenome. Genetics. 2016;204(4):1613–26. 10.1534/genetics.116.193227. 27794028 10.1534/genetics.116.193227 PMC5161289

[59] Stull GW, Qu XJ, Parins-Fukuchi C, Yang YY, Yang JB, Yang ZY, Hu Y, Ma H, Soltis PS, Soltis DE, et al. Gene duplications and phylogenomic conflict underlie major pulses of phenotypic evolution in gymnosperms. Nat Plants. 2021;7(8):1015–25. 10.1038/s41477-021-00964-4. 34282286 10.1038/s41477-021-00964-4

[60] Wu JJ, Han YW, Lin CF, Cai J, Zhao YP. Benchmarking gene set of gymnosperms for assessing genome and annotation completeness in BUSCO. Hortic Res. 2023;10(9):uhad165. 10.1093/hr/uhad165. 37731863 10.1093/hr/uhad165 PMC10508034

[61] Guan D, McCarthy SA, Wood J, Howe K, Wang Y, Durbin R. Identifying and removing haplotypic duplication in primary genome assemblies. Bioinformatics. 2020;36(9):2896–8. 10.1093/bioinformatics/btaa025. 31971576 10.1093/bioinformatics/btaa025 PMC7203741

[62] Brunner AM, Varkonyi-Gasic E, Jones RC. Phase change and phenology in trees. In: *Comparative and evolutionary genomics of angiosperm trees.* Edited by Groover A, Cronk Q, vol. 21. Cham: Springer International Publishing; 2017: 227–274.

[63] Hu L, Yang L. Time to fight: molecular mechanisms of age-related resistance. Phytopathology. 2019;109(9):1500–8. 10.1094/PHYTO-11-18-0443-RVW. 31192748 10.1094/PHYTO-11-18-0443-RVW

[64] Murshid A, Eguchi T, Calderwood SK. Stress proteins in aging and life span. Int J Hyperth. 2013;29(5):442–7. 10.3109/02656736.2013.798873. 10.3109/02656736.2013.798873 PMC4083487 23742046

[65] Ohama N, Sato H, Shinozaki K, Yamaguchi-Shinozaki K. Transcriptional regulatory network of plant heat stress response. Trends Plant Sci. 2017;22(1):53–65. 10.1016/j.tplants.2016.08.015. 27666516 10.1016/j.tplants.2016.08.015

[66] Ye Z-L, Liu J-Y, Feng J, Li W-F. Transcriptome analysis provides insights into Korean pine tree aging and response to shading. Forests. 2024;15(2):291. 10.3390/f15020291.

[67] Hu Y, Liang W, Yin C, Yang X, Ping B, Li A, Jia R, Chen M, Luo Z, Cai Q, et al. Interactions of *OsMADS1* with floral homeotic genes in rice flower development. Mol Plant. 2015;8(9):1366–84. 10.1016/j.molp.2015.04.009. 25917758 10.1016/j.molp.2015.04.009

[68] Reinheimer R, Kellogg EA. Evolution of *AGL6-like* MADS box genes in grasses (Poaceae): ovule expression is ancient and palea expression is new. Plant Cell. 2009;21(9):2591–605. 10.1105/tpc.109.068239. 19749151 10.1105/tpc.109.068239 PMC2768931

[69] Li H, Liang W, Jia R, Yin C, Zong J, Kong H, Zhang D. The *AGL6-like* gene *OsMADS6* regulates floral organ and meristem identities in rice. Cell Res. 2010;20(3):299–313. 10.1038/cr.2009.143. 20038961 10.1038/cr.2009.143

[70] Li H, Liang W, Hu Y, Zhu L, Yin C, Xu J, Dreni L, Kater MM, Zhang D. Rice MADS6 interacts with the floral homeotic genes SUPERWOMAN1, MADS3, MADS58, MADS13, and DROOPING LEAF in specifying floral organ identities and meristem fate. Plant Cell. 2011;23(7):2536–52. 10.1105/tpc.111.087262. 21784949 10.1105/tpc.111.087262 PMC3226212

[71] Yoo SK, Wu X, Lee JS, Ahn JH. *AGAMOUS-LIKE 6* is a floral promoter that negatively regulates the *FLC/MAF* clade genes and positively regulates *FT* in *Arabidopsis*. Plant J. 2011;65(1):62–76. 10.1111/j.1365-313X.2010.04402.x. 21175890 10.1111/j.1365-313X.2010.04402.x

[72] Wager A, Browse J. Social network: JAZ protein interactions expand our knowledge of jasmonate signaling. Front Plant Sci. 2012;3:41. 10.3389/fpls.2012.00041. 22629274 10.3389/fpls.2012.00041 PMC3355530

[73] Xia W, Yu H, Cao P, Luo J, Wang N. Identification of TIFY family genes and analysis of their expression profiles in response to phytohormone treatments and *Melampsora larici-populina* infection in Poplar. Front Plant Sci. 2017;8:493. 10.3389/fpls.2017.00493. 28424731 10.3389/fpls.2017.00493 PMC5380741

[74] Guan Y, Ding L, Jiang J, Shentu Y, Zhao W, Zhao K, Zhang X, Song A, Chen S, Chen F. Overexpression of the *CmJAZ1-like* gene delays flowering in *Chrysanthemum morifolium*. Hortic Res. 2021;8(1):87. 10.1038/s41438-021-00525-y. 33795661 10.1038/s41438-021-00525-y PMC8016864

[75] Gomes GLB, Scortecci KC. Auxin and its role in plant development: structure, signalling, regulation and response mechanisms. Plant Biol (Stuttg). 2021;23(6):894–904. 10.1111/plb.13303. 34396657 10.1111/plb.13303

[76] Traversari S, Giovannelli A, Emiliani G. Wood formation under changing environment: omics approaches to elucidate the mechanisms driving the early-to-latewood transition in conifers. Forests. 2022;13(4):608. 10.3390/f13040608.

[77] Imran QM, Falak N, Hussain A, Mun BG, Sharma A, Lee SU, Kim KM, Yun BW. Nitric oxide responsive heavy metal-associated gene *AtHMAD1* contributes to development and disease resistance in *Arabidopsis Thaliana*. Front Plant Sci. 2016;7:1712. 10.3389/fpls.2016.01712. 27917181 10.3389/fpls.2016.01712 PMC5116471

[78] Janusz G, Pawlik A, Swiderska-Burek U, Polak J, Sulej J, Jarosz-Wilkolazka A, Paszczynski A. Laccase properties, physiological functions, and evolution. Int J Mol Sci. 2020;21(3):966. 10.3390/ijms21030966. 32024019 10.3390/ijms21030966 PMC7036934

[79] Xu T, Wang J, Li C, Zhang Y, Zhang Z, Kang X, Yang J. *EuMYB308* regulates lignin accumulation by targeting *EuLAC17* in *Eucalyptus urophylla*. Ind Crops Prod. 2024;218:118988. 10.1016/j.indcrop.2024.118988.

[80] Wang Y, Yu W, Ran L, Chen Z, Wang C, Dou Y, Qin Y, Suo Q, Li Y, Zeng J, et al. *DELLA-NAC* interactions mediate GA signaling to promote secondary cell wall formation in cotton stem. Front Plant Sci. 2021;12:655127. 10.3389/fpls.2021.655127. 34305962 10.3389/fpls.2021.655127 PMC8299300

[81] Wei S, Zhang W, Fu R, Zhang Y. Genome-wide characterization of 2-oxoglutarate and Fe(II)-dependent dioxygenase family genes in tomato during growth cycle and their roles in metabolism. BMC Genomics. 2021;22(1):126. 10.1186/s12864-021-07434-3. 33602133 10.1186/s12864-021-07434-3 PMC7891033

