# Supplementary material for: Spatiotemporal dynamics of age-related genes and the regulatory network of LaAGL2-3 in Larix kaempferi (Lamb.) Carr. based on the latest genome annotation
Source: BMC Plant Biol. 2025 Dec 15;26:99. doi: 10.1186/s12870-025-07956-y (PMC12821181; doi:10.1186/s12870-025-07956-y)
Supplement: Supplementary file 1 — Supplementary Material 1: Figure S1-S2. [file 12870_2025_7956_MOESM1_ESM.docx]

# Figure S1


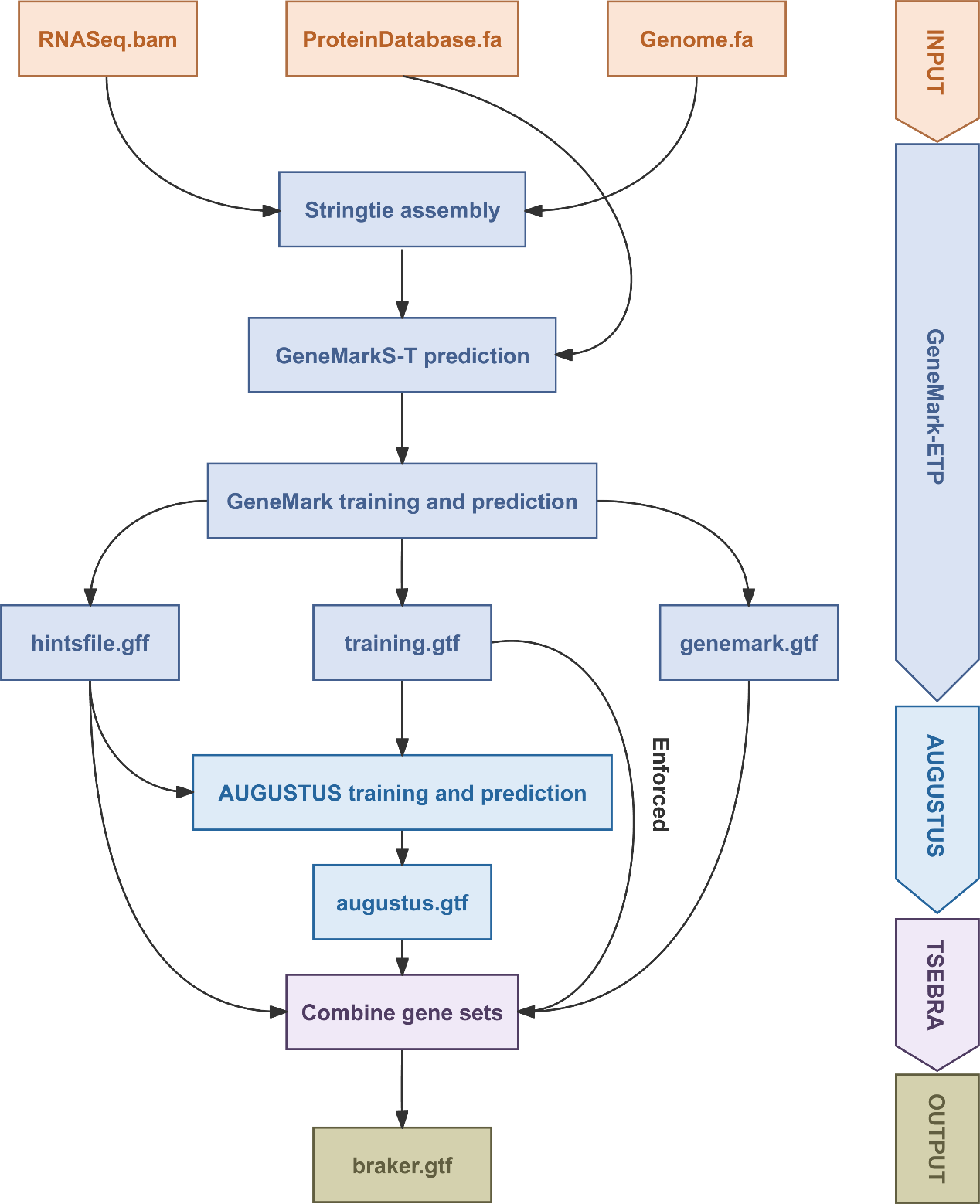


**Figure S1** BRAKER 3 genome structure annotation pipeline. The flowchart is from BRAKER3 project (<https://github.com/Gaius-Augustus/BRAKER>).

# Figure S2


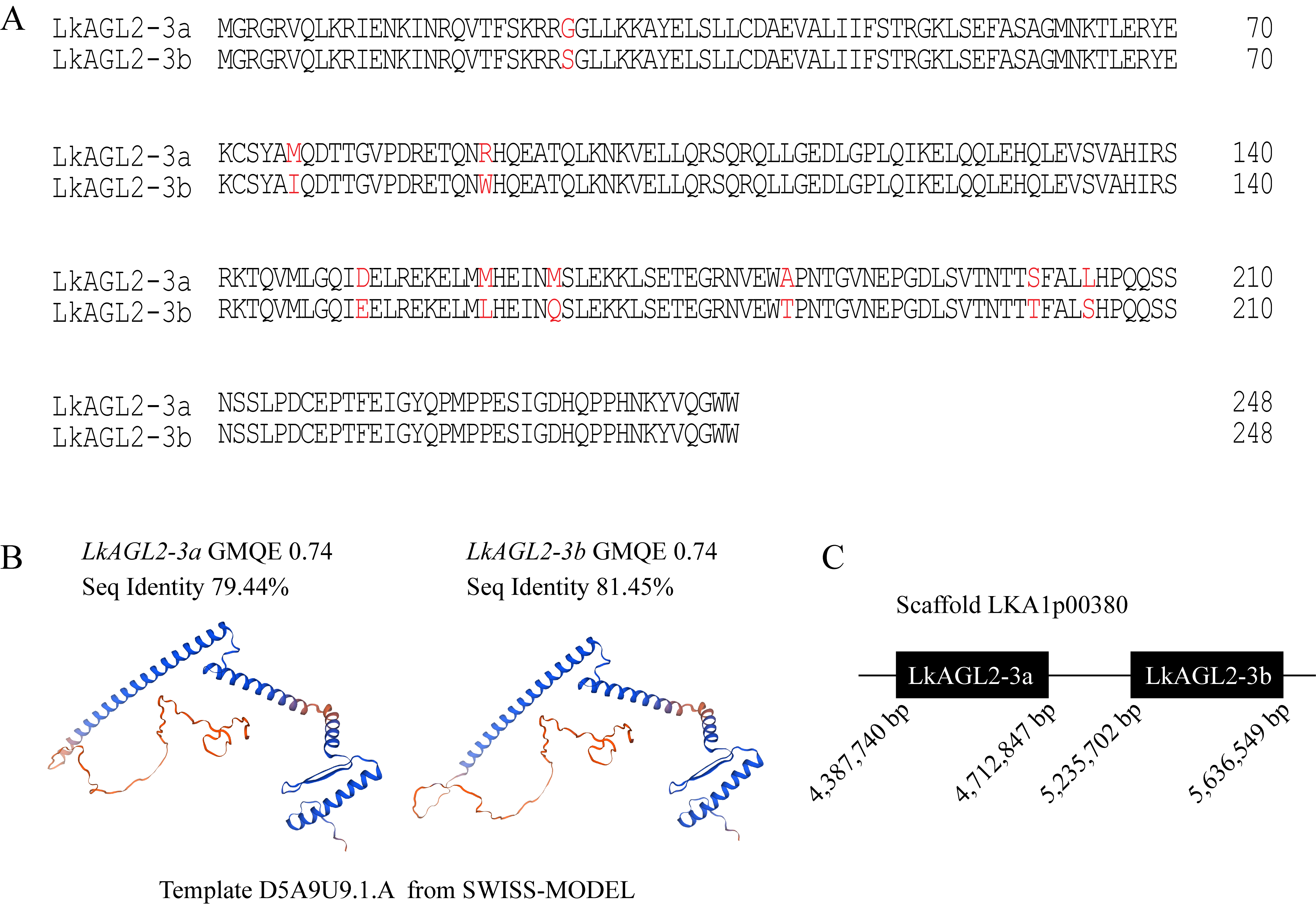


**Figure S2.** The differences between LkAGL2-3a and LkAGL2-3b.

A: The alignment of LkAGL2-3a and LkAGL2-3b. Only 9 amino acids are different. Character in red is the different amino acids.

B: The predicted tertiary structure of LkAGL2-3a and LkAGL2-3b. GMQE: global model quality estimate. Seq Identity: sequence identity of AlphaFold DB model of D5A9U9_PICSI.

C: The position of *LkAGL2-3a* and *LkAGL2-3b* in the genome.
